# Oxidative Transformations of 3,4-Dihydroxyphenylacetaldehyde Generate Potential Reactive Intermediates as Causative Agents for Its Neurotoxicity

**DOI:** 10.3390/ijms222111751

**Published:** 2021-10-29

**Authors:** Shosuke Ito, Hitomi Tanaka, Makoto Ojika, Kazumasa Wakamatsu, Manickam Sugumaran

**Affiliations:** 1 Institute for Melanin Chemistry, Fujita Health University, Toyoake 470-1192, Aichi, Japan; hitanaka@u-gifu-ms.ac.jp (H.T.); kwaka@fujita-hu.ac.jp (K.W.); 2 Department of Medical Technology, School of Health Sciences, Gifu University of Medical Science, Seki 501-3892, Gifu, Japan; 3 Department of Applied Biosciences, Graduate School of Bioagricultural Sciences, Nagoya University, Nagoya 464-8601, Aichi, Japan; ojika@agr.nagoya-u.ac.jp; 4 Department of Biology, University of Massachusetts Boston, Boston, MA 02125, USA; Manickam.sugumaran@umb.edu

**Keywords:** 3,4-dihydroxyphenylacetaldehyde (DOPAL), 3,4-dihydroxyphenylglycolaldehyde (DOPEGAL), Parkinson’s disease, tyrosinase, *ortho*-quinone, quinone methide

## Abstract

Neurogenerative diseases, such as Parkinson’s disease, are associated, not only with the selective loss of dopamine (DA), but also with the accumulation of reactive catechol-aldehyde, 3,4-dihydroxyphenylacetaldehyde (DOPAL), which is formed as the immediate oxidation product of cytoplasmic DA by monoamine oxidase. DOPAL is well known to exhibit toxic effects on neuronal cells. Both catecholic and aldehyde groups seem to be associated with the neurotoxicity of DOPAL. However, the exact cause of toxicity caused by this compound remains unknown. Since the reactivity of DOPAL could be attributed to its immediate oxidation product, DOPAL-quinone, we examined the potential reactions of this toxic metabolite. The oxidation of DOPAL by mushroom tyrosinase at pH 5.3 produced conventional DOPAL-quinone, but oxidation at pH 7.4 produced the tautomeric quinone-methide, which gave rise to 3,4-dihydroxyphenylglycolaldehyde and 3,4-dihydroxybenzaldehyde as products through a series of reactions. When the oxidation reaction was performed in the presence of ascorbic acid, two additional products were detected, which were tentatively identified as the cyclized products, 5,6-dihydroxybenzofuran and 3,5,6-trihydroxybenzofuran. Physiological concentrations of Cu(II) ions could also cause the oxidation of DOPAL to DOPAL-quinone. DOPAL-quinone exhibited reactivity towards the cysteine residues of serum albumin. DOPAL-oligomer, the oxidation product of DOPAL, exhibited pro-oxidant activity oxidizing GSH to GSSG and producing hydrogen peroxide. These results indicate that DOPAL-quinone generates several toxic compounds that could augment the neurotoxicity of DOPAL.

## 1. Introduction

Neurogenerative diseases, such as Parkinson’s disease (PD), are associated typically with the selective loss of dopamine (DA). However, 3,4-dihydroxyphenylaldehyde (DOPAL), which is formed as the immediate oxidation product of cytoplasmic DA by monoamine oxidase (MAO) in the outer mitochondrial membrane, has also been attributed to the cause of disease [1,2,3]. DOPAL is well known to exhibit toxic effects on neuronal cells at concentrations as low as 7 µM [4], which is not far from the physiological concentration of 2 µM DOPAL in dopaminergic cells [5,6]. DOPAL is also toxic to substantia nigral neurons in vivo [7,8]. The DOPAL to DA ratio is increased 3-fold in the putamen of PD patients compared to controls [2,3]. Thus, the accumulation of DOPAL might contribute to neuronal malfunctions and eventual loss of dopaminergic neurons, leading to neurogenerative diseases [7,9,10,11].

**Figure 1:**
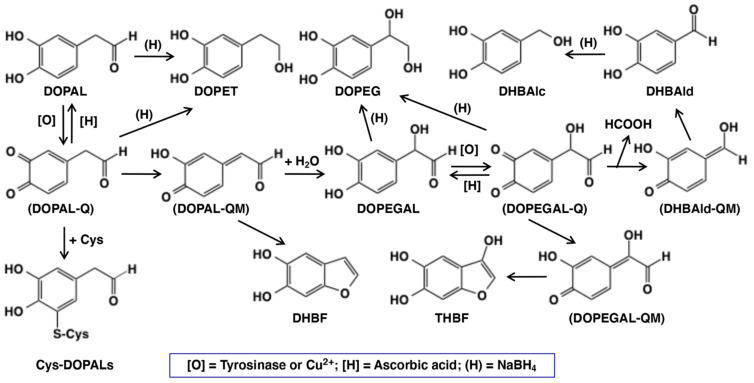
Scheme showing the oxidative fate of DOPAL by tyrosinase or copper(II) ions in the absence or presence of ascorbic acid [H] or cysteine (Cys). Other than DOPAL-Q, all other products were identified in the present study. Abbreviation of the compounds are: Cys—L-cysteine; Cys-DOPALs—cysteine adducts of DOPAL-Q; DHBAlc—3,4-dihydroxybenzylalcohol; DHBAld—3,4-dihydroxybenzaldehyde; DHBF—5,6-dihydroxybenzofuran; DOMA—3,4-dihydroxymandelic acid; DOPAC—3,4-dihydroxyphenylacetic acid; DOPAL—3,4-dihydroxyphenylacetaldehyde; DOPEGAL—3,4-dihydroxyphenylglycolaldehyde; DOPET—2-(3,4-dihydroxyphenyl)ethanol; DOPEG—3,4-dihydroxyphenylethylene glycol; [O]—tyrosinase or copper(II) ions; Q—quinone; QM—quinone methide; (H)—sodium borohydride; and THBF—3,5,6-trihydroxybenzofuran. Compounds in parenthesis are intermediates with extremely high reactivity and cannot be isolated.

The possible mechanisms of neurotoxicity of DOPAL have been studied by a few groups in recent years. DOPAL possesses two chemically reactive functional groups, viz., catecholic and aldehyde groups (Figure 1). Both groups appear to be involved in the neurotoxicity of DOPAL [12], which leads to the proposal of the so-called “catechol-aldehyde” hypothesis [13,14,15]. The aldehyde group can form a Schiff’s base with the free primary amino group of proteins, and would account for their major role in binding with neuronal proteins, such as α-synuclein, with the participation of a lysine side chain [5,10,16,17,18]. On the other hand, the catecholic group will participate in protein modification, depletion of cellular thiols, etc., through its primary oxidation product, DOPAL-quinone, much like many other quinones that are reportedly involved in the formation of protein adducts and even cross links [19,20]. In support of this proposition, *O*-methylation of DOPAL, which prevents DOPAL-quinone formation, has been shown to greatly attenuate its toxicity to dopaminergic cells [21]. Furthermore, addition of *N*-acetylcysteine mitigates such protein modifications, confirming the crucial reactivities of the quinonoid form of the catechol group [12]. Therefore, it is highly probable that DOPAL-quinone and its metabolites play a crucial role in the toxicity of DOPAL. Preliminary studies conducted by two groups [21,22] indicated the high reactivity of DOPAL-quinone. Since little information is available on the intricate reactions of DOPAL-quinone, we decided to perform a detailed study on the potential reactions of this toxic metabolite. Our results indicate that the immediate two electron oxidation product, DOPAL-quinone, exhibits unusual reactivities and generates several toxic compounds that could augment the neurotoxicity of DOPAL.

## 2. Results

### 2.1. Oxidative Transformation of DOPAL by Tyrosinase-Catalyzed Reaction

**Figure 2:**
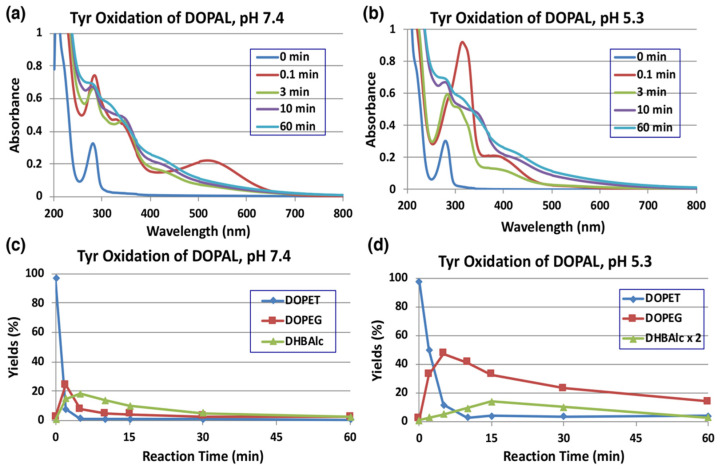
Time course of the tyrosinase-catalyzed oxidation of DOPAL (100 µM) and HPLC analysis of reaction products. (**a**) UV/visible spectral changes of DOPAL at pH 7.4 and 25 °C. (**b**) At pH 5.3 and 25 °C. (**c**) HPLC analysis following the tyrosinase-catalyzed oxidation of DOPAL at pH 7.4 and 37 °C and (**d**) at pH 5.3 and 37 °C. Reaction was stopped by the addition of NaBH_4_ followed by 0.8 M HClO_4_. Data for the spectral changes were obtained from single experiments, but reproducibility was confirmed for each experiment (**a**,**b**). Data for the HPLC analysis were obtained from averages of two independent experiments (**c**,**d**).

Tyrosinase is an enzyme oxidizing a variety of *p*-substituted phenols and their catechol derivatives to their corresponding *o*-quinones [19,23,24,25,26,27]. Accordingly, DOPAL also readily serves as a substrate for the commercially available mushroom tyrosinase. The immediate oxidation product of catechols by tyrosinase is the corresponding *o*-quinone. Though simple *o*-quinones are reactive and exhibit various nucleophilic reactivities, often in a short time; their formation and accumulation in reaction mixture can easily be assessed by visible spectroscopy, as they tend to exhibit absorbance in the visible region at about 400 nm. 3,4-Dihydroxyphenylacetic acid (DOPAC)-quinone, the oxidized product of DOPAL-quinone (aldehyde to acid), for example, is readily formed by the oxidation of DOPAC with mushroom tyrosinase and exhibits typical visible absorbance maximum at 400 nm [25]. The time course of the spectral changes associated with the oxidation of DOPAL (100 µM) by tyrosinase at pH 7.4 shown in Figure 2a, on the other hand, indicates the formation of a product exhibiting absorbance maximum at 518 nm, which was puzzling. This compound readily and rapidly decayed in the reaction mixture with time to give featureless spectra, indicating a probable polymerization reaction. The same observation was also earlier reported by Anderson et al. [22], who suggested that the 520 nm absorption peak is a semiquinone radical and it undergoes dismutation, producing DOPAL and DOPAL-quinone. Such dismutation has been reported for laccase, as laccase oxidizes catechols by one electron oxidation to semiquinones, which rapidly dismutates in solution to parent catechols and two electron oxidized product—quinones [28]. However, tyrosinase is not known to cause one electron oxidation of any of the related compounds. In the case of 1,2-dehydro-*N*-acetyldopamine (dehydro-NADA), the immediate detectable product of two electron oxidation of dehydro-NADA by tyrosinase is a quinone methide exhibiting absorbance at 485 nm at neutral pH values. This quinone methide, however, is converted to conventional quinone product at more acidic pH values [26,29,30], as quinone to quinone methide tautomerization is a base catalyzed reaction [20,30,31,32].

Thus, we attempted tyrosinase-catalyzed oxidation of DOPAL at pH 5.3 instead of 7.4. As shown in Figure 2b, reaction of DOPAL with tyrosinase produced the typical *o*-quinone, exhibiting absorbance at about 390 nm. Again, the transiently formed DOPAL-quinone seemed to be unstable and readily exhibited further reactions, as evidenced by the fast spectral changes occurring during the reaction. It appears likely that DOPAL-*o*-quinone is very rapidly tautomerized to DOPAL-quinone methide at pH 7.4, as compared to the slower reaction at pH 5.3 like other 4-alkylsubstituted quinones [20,30,31,32]. Support for the quinone methide of DOPAL having a 518 nm absorption comes from the two-electron oxidation of DOPAL by sodium periodate (NaIO_4_). When 100 µM DOPAL was oxidized by 100 µM sodium periodate at pH 7.4, an immediately detectable chromophore, which is almost identical to that in Figure 2a, appeared in the reaction mixture (Figure S1). Since periodate is also a two-electron oxidant like that of tyrosinase, it was concluded that the product formed in this reaction mixture is indeed quinone methide and not a semiquinone. Once formed, quinone methide can exhibit a variety of reactions through its side chain. Interestingly, the related DOPAC-quinone methide was also shown to be extremely unstable exhibiting rapid reactivities [27,33]. In this case, formation of 3,4-dihydroxybenzaldehyde (DHBAld), as well as cyclized product of DOPAC-quinone—2,5,6-trihydroxybenzofuran, were identified as the immediate products [27]. Further studies indicated that the cyclized product was very unstable and exhibited polymerization reaction [27,33]. A similar reaction is also possible with DOPAL-quinone, hence we conducted further studies.

As the UV/visible spectral changes appeared complex, the metabolic fate of DOPAL-quinone was examined by analyzing the reaction mixture using HPLC. Since *o*-quinones are highly reactive and unstable, we reduced the quinonoid products formed in the reaction mixture back to catechols using NaBH_4_ for easy detection by HPLC by monitoring the absorbance at 280 nm. NaBH_4_ also reduces the aldehyde group to alcohol; hence, any product containing this functional group will be converted to a stable alcohol. We have previously used the reduction of carbonyl group with NaBH_4_ for tyrosinase-catalyzed oxidation of the leukoderma-inducing agent, raspberry ketone [34]. The reduction also stabilizes aldehydes and prevents any further undesired reactions. DOPAL-quinone will be thus reduced to 3,4-dihydroxyphenylethanol (DOPET). Figure 2c shows the time course of DOPAL-quinone decay at pH 7.4 after NaBH_4_ reduction. DOPAL-quinone (detected in the form of DOPET) disappeared rapidly from the reaction mixture to give two new products. Taking into account of the chemical reactivity of *o*-quinones [19], these two products were identified as 3,4-dihydroxyphenylethylene glycol (DOPEG) and 3,4-dihydroxybenzylalcohol (DHBAlc). Their identities were further confirmed by co-injection with the authentic samples with HPLC. The mechanism of production of DOPEG and DHBAlc can be explained as follows (Figure 1). DOPAL-quinone first gets isomerized to DOPAL-quinone methide, as observed with several 4-alkyl substituted quinones [19,20,23,26,27,29,33,35]. 1,6-Water addition to DOPAL-quinone methide produces 3,4-dihydroxyphenylglycolaldehyde (DOPEGAL), which is a known deamination product of norepinephrine (NE) [36]. The *o*-quinone of DOPEGAL is then transformed to DHBAld by a deformylation reaction. The occurrence of such deformylation reactions has been well documented in the literature [37]. The NaBH_4_ reduction of DOPEGAL and DHBAld yields DOPEG and DHBAlc, respectively, which were detected as the end products. DHBAld also decayed rapidly to form ill-defined products. These oxidative transformations were slowed down by performing the oxidation at pH 5.3 (Figure 2d). Under these conditions, DOPAL-quinone decayed with a half-life of ca. 2 min and DOPEG was produced in much higher yields (47% at 5 min) than at pH 7.4 (24% at 2 min). The lower yield of DOPEG at a higher pH suggests the presence of another route of transformation for DOPAL-quinone, such as the production of extremely reactive intermediates that cannot be detected under oxidative conditions (see below).

### 2.2. Oxidative Transformation of DOPAL by Tyrosinase-Catalyzed Reaction in the Presence of Ascorbic Acid (AA)

**Figure 3:**
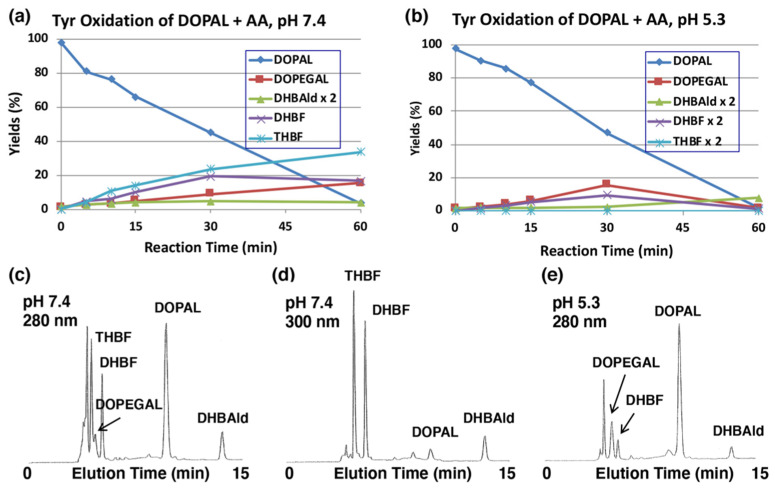
Time course of the tyrosinase-catalyzed oxidation of DOPAL (100 µM) in the presence of AA (1 mM). (**a**) HPLC analysis following the tyrosinase-catalyzed oxidation of DOPAL plus AA at pH 7.4 and 37 °C and (**b**) at pH 5.3 and 37 °C. (**c**,**d**) HPLC chromatograms of the tyrosinase-catalyzed oxidation of DOPAL plus AA at pH 7.4 after 30 min, detected at 280 nm (**c**) and at 300 nm (**d**). (**e**) pH 5.3 after 30 min, detected at 280 nm. Reaction was stopped by the addition of 0.8 M HClO_4_. Data for the HPLC analysis were obtained from averages of two independent determinations (**a**,**b**).

The production of DOPEGAL and DHBAld during tyrosinase-catalyzed oxidation of DOPAL was confirmed by reducing *o*-quinones to catechols using ascorbic acid (AA), which is known to reduce *o*-quinones to catechols, but not the aldehyde group. Tyrosinase-catalyzed oxidation was performed in the presence of 1 mM AA (10 mol eq.). As shown in Figure 3a, the production of DOPEGAL (9% at 30 min) and DHBAld (2% at 30 min) were in fact observed. Their identification was confirmed by co-injection with the authentic samples using HPLC. When the oxidation was performed at pH 5.3, the yield of DOPEGAL was increased to 16% at 30 min (Figure 3b). The decrease of DOPEGAL at 60 min could be due to a possible consumption of all the AA.

We could also observe the production of two new intermediates in yields higher than DOPEGAL, as shown on HPLC chromatogram at pH 7.4 (Figure 3c). These two products are more prominent when HPLC eluents were monitored at 300 nm (Figure 3d). In fact, UV spectra of those intermediates exhibited peaks around 300 nm (Figure S2), suggesting that they are not simple catecholic compounds like DOPAL and DOPEGAL. We propose that those two products are 5,6-dihydroxybenzofuran (DHBF) and 3,5,6-trihydroxybenzofuran (THBF) (Figure 1). This generalization is in the same line as that of the reaction of DOPAC-quinone/quinone methide which exhibits rapid intramolecular cyclization yielding 2,5,6-trihydroxybenzofuran [27,33]. At pH 7.4, the yields of DHBF and THBF were higher, 19% and 24% (30 min), respectively, compared to the 9% yield of DOPEGAL. On the other hand, at pH 5.3, the yields of DHBF and THBF were much lower, 5% and <1%, respectively, compared to the 16% yield of DOPEGAL (Figure 3b). Apparently, a higher pH favors the production of DHBF and THBF, while a lower pH favors the production of DOPEGAL.

### 2.3. Copper (II)-Catalyzed Oxidation of DOPAL Produces DOPEGAL and DHBAld

**Figure 4:**
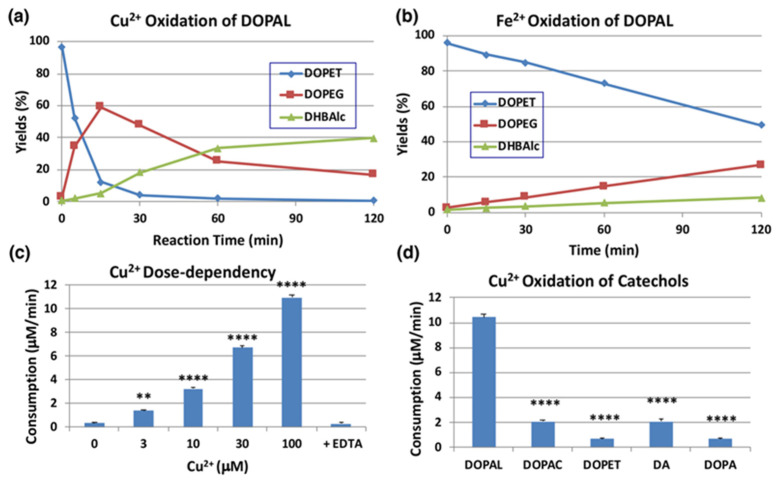
Time course of HPLC following of the metal-catalyzed oxidation of DOPAL (100 µM). (**a**) Cu^2+^ (100 µM) at pH 7.4 and 37 °C. (**b**) Fe^2+^ (100 µM) at pH 7.4 and 37 °C. Reaction was stopped by the addition of NaBH_4_ followed by 0.8 M HClO_4_. Data were obtained from averages of two independent experiments (**a**,**b**). (**c**) Dose dependency of the effect of Cu^2+^ ions. EDTA (200 µM) was added to Cu^2+^ (100 µM). Reaction was stopped after 5-min reaction for 30 and 100 µM Cu^2+^, after 15-min for 10 µM Cu^2+^, and after 30-min for the rest. The consumption rates (µM/min) were calculated by dividing the consumption (µM) by the reaction time. ** *p* < 0.01 between 0 µM and 3 µM Cu^2+^ and **** *p* < 0.0001 between 0 µM Cu^2+^ and 10 to 100 µM Cu^2+^. (**d**) Comparison of reactivity among relevant catechols (100 µM) with Cu^2+^ (100 µM). Reaction was stopped after 15 min (5 min for DOPAL) reaction at pH 7.4 and 37 °C by the addition of NaBH_4_ followed by 0.8 M HClO_4_. **** *p* < 0.0001 between DOPAL and other catecholamines. Mean ± SEM from three independent experiments (**c**,**d**).

We next examined the effects of transition metal ions of biological significance, such as Cu^2+^ and Fe^2+^ (100 µM). As shown in Figure 4a, Cu^2+^ ions oxidized DOPAL rapidly with nearly 90% consumption at pH 7.4 within 15 min. The major products identified in the reaction mixture were DOPEG and DHBAlc after NaBH_4_ reduction, as have been observed for the tyrosinase-catalyzed oxidation. On the other hand, the effects of Fe^2+^ ions were minimal (50% consumption of DOPAL at 120 min; Figure 4b). Fe^3+^ (100 µM) showed even weaker effects than Fe^2+^ (43% consumption of DOPAL) with minimal differences from the control, which was autoxidation (30% consumption, Figure S3a,b). Our finding that Cu^2+^ ions but not Fe^3+^ ions oxidize DOPAL is in similar line to the observation made by Jinsmaa et al. [38] that Cu^2+^ (and to a much lesser extent Fe^2+^) ions augment DOPAL-induced oligomerization of α-synuclein but Fe^3+^ and Cu+ could not. We then examined the dose-dependency of Cu^2+^ ions (Figure 4c). Cu^2+^ ions oxidized DOPAL in a dose-dependent manner. Even at concentration as low as 3 µM, it significantly oxidized DOPAL. Such oxidation was completely suppressed by the inclusion of excess of EDTA in the reaction mixture thus providing pivotal evidence for the Cu^2+^ ion assisted oxidation of DOPAL.

We then compared the reactivity of several biologically relevant catechols, such as DOPA, DA, DOPAC, and DOPET, towards Cu^2+^ ions. DA is the precursor of DOPAL in catecholamine metabolism, while DOPAC and DOPET are oxidized and reduced metabolites of DOPAL, catalyzed by aldehyde dehydrogenase and aldehyde reductase, respectively [39]. As shown in Figure 4d, DOPAL was ca. 5-fold more reactive than DOPAC and DA and >10-fold more reactive than DOPET and DOPA at pH 7.4. It is likely that DOPAL is the most reactive among biological catechols toward Cu^2+^-catalyzed oxidation due to the presence of an electron withdrawing aldehyde group.

### 2.4. Reaction of DOPAL-Q with L-Cysteine (Cys) and Thiol-Proteins

**Figure 5:**
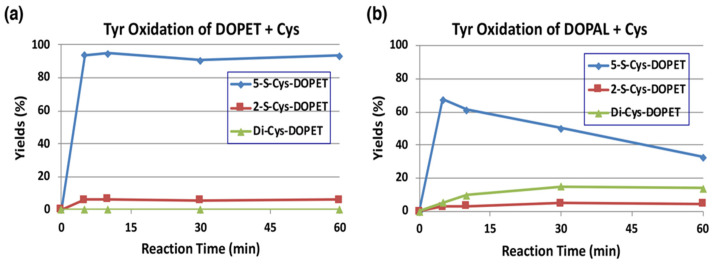
Time course of HPLC following of the tyrosinase-catalyzed oxidation of DOPET or DOPAL (100 µM) in the presence of Cys (200 µM) at pH 7.4 and 37 °C. (**a**) DOPET. (**b**) DOPAL. Reaction was stopped by the addition of NaBH_4_ followed by 0.8 M HClO_4_. Data were obtained from averages of two independent experiments (**a**,**b**).

The reaction of *o*-quinones with thiols to give thiolated catechols is implicated, not only in the protection against cytotoxic *o*-quinone by simple thiols, but is also encountered in the cytotoxicity exhibited by quinones due to binding to proteins through the cysteinyl residues in cellular proteins [19]. Tyrosinase-catalyzed oxidation of DOPAL was examined in the presence of 2 mol eq. of cysteine (Cys) at pH 7.4. The reaction was terminated by reduction with NaBH_4_. To make the identification of the Cys adducts straightforward, we first examined tyrosinase-catalyzed oxidation of DOPET in the presence of Cys. As shown in Figure 5a, 5-*S*-Cys-DOPET and 2-*S*-Cys-DOPET were produced rapidly in a ratio of ca. 20:1 and remained stable during 60-min reaction. Di-adduct was not detected. On the other hand, the oxidation of DOPAL in the presence of Cys afforded a 15% yield of di-Cys-DOPET (assumed to be 2,5-*S*,*S*-diCys-DOPET) in addition to 5-*S*-Cys-DOPET and 2-*S*-Cys-DOPET in a ratio of ca. 20:1 after NaBH_4_ reduction (Figure 5b). The 15% yield of the di-adduct was surprising because the yield is much higher than 4–5% yield of di-Cys-DOPA and di-Cys-DA [40,41]. The production of di-Cys adduct suggests that 5-*S*-Cys-DOPAL is oxidized by tyrosinase to the *o*-quinone form.

**Figure 6:**
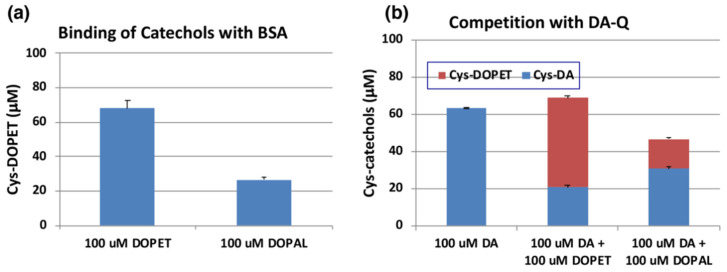
(**a**) Tyrosinase-catalyzed binding of DOPET and DOPAL (100 µM) with BSA (300 µM). (**b**) Competition of tyrosinase-catalyzed binding of DA (100 µM) with DOPET or DOPAL (100 µM). Mean ± SEM from three independent experiments (**a**,**b**).

We next examined the reactivity of DOPAL-quinone with thiol proteins using the similar conditions as outlined above. At the end of the reaction, the entire reaction mixture was subjected to HCl hydrolysis in the presence of thioglycolic acid and phenol (to protect the catecholic products) followed by alumina extraction of Cys-catechol products and HPLC analysis [42,43]. We had to perform additional control experiments with standards as we encountered undesired substitution reactions. One problem that we had to overcome, is substitution of hydroxyl group in the side chain of DOPET with chlorine atom in HCl. This side reaction was discovered with 4-(4-hydroxyphenyl)-2-hydroxybutane, rhododendrol (RD) [44]. Another unwanted reaction is substitution of carboxymethylthio (-SCH_2_COOH) group with the hydroxyl group, which was unraveled when NE was subjected to the HCl hydrolysis in presence of thioglycolic acid [45]. To check for these possibilities and monitor only the addition of quinone to the thiol groups, we performed two reactions. The first reaction had tyrosinase, DOPET and 3 eq. bovine serum albumin (BSA) which contained 0.30 mol cysteine residue/mol BSA [46]. The resultant adduct was hydrolyzed with 6 M HCl containing 5% thioglycolic acid and 1% phenol and the products analyzed by HPLC. The second reaction containing the standard Cys-DOPET was also subjected to the same HCl hydrolysis treatment. Both the reactions afforded three major products. To determine their structure, the HCl hydrolysis of Cys-DOPET was performed in a preparative scale and the products subjected to preparative HPLC. The fast-moving product was identified as the unreacted Cys-DOPET. The second product was identified as the carboxymethylation derivative by ^1^H-NMR and mass spectral analysis [45]. The third, slow-moving product was characterized as the chloro derivative of Cys-DOPET [44]. Comparison of the yields of these three products in control and experiment indicated that the binding of the cysteine residue of BSA to DOPET-quinone was 68% (Figure 6a). On the other hand, when DOPAL was oxidized by tyrosinase in the presence of 3 eq. BSA and reduced with NaBH_4_, the yield of Cys-DOPET-derived products was dropped to 27% (Figure 6a).

The yield of DOPAL-BSA conjugates was much lower than that of DOPET-BSA. One possible reason may be ascribed to the high tendency of DOPAL-quinone for isomerization to quinone methide tautomer (Figure 1). In fact, several products appeared in the HCl hydrolysates of the reaction between DOPAL-quinone with BSA. However, attempts to identify the binding products of the quinone methide with BSA were unsuccessful.

We then compared the reactivity of *o*-quinone products from DOPET and DOPAL against that of DA. Figure 6b indicates that oxidation of 100 µM of DA in the presence of 3 eq. BSA afforded 63% yield of Cys-DA isomers after HCl hydrolysis. When 100 µM of DOPET or DOPAL was present in addition to 100 µM of DA, the yield of Cys-DA isomers was decreased to 21% or 31%, respectively. These decreases can be ascribed to the competition between DA-quinone and DOPET-quinone or DOPAL-quinone, respectively. Based on the relative ratio of Cys-DA isomers from (DA+DOPET)/DA and (DA+DOPAL)/DA of 0.33 and 0.49, the relative reactivity of *o*-quinones toward the Cys residue of BSA can be inferred that DOPET-quinone is 2.0-fold more reactive than DA-quinone, while DOPAL-quinone is as reactive as DA-quinone. This interpretation was supported by the yields of Cys-DOPET and Cys-DA isomers from oxidation products of 100 µM DA + 100 µM DOPET with BSA to be 48% and 21%, respectively, giving a ratio of Cys-DOPET/Cys-DA of 2.3 (Figure 6b).

### 2.5. Pro-Oxidant Activity of DOPAL-Oligomer

We have recently shown that tyrosinase-catalyzed oxidation products (oligomers) of RD, raspberry ketone, resveratrol, and equol exhibit potent pro-oxidant activity, as assessed by the oxidation of GSH to GSSG and the production of hydrogen peroxide [34,46,47,48]. RD is well-known as the causative agent of leucoderma in nearly 20,000 consumers who used RD-containing skin whitening cosmetics [49]. Because of the presence of reactive catechol and aldehyde groups, we expected that DOPAL-oligomer would also possess potent pro-oxidant activity. Initial studies aimed at HPLC detection of reaction products as performed in the case of RD and other phenolic compounds [34,46,47], failed due to high tendency of adsorption of DOPAL (or DOPET after reduction) and its oxidation products onto the HPLC matrix. Therefore, we abandoned this approach and directly used the reaction product for the following experiments.

**Figure 7:**
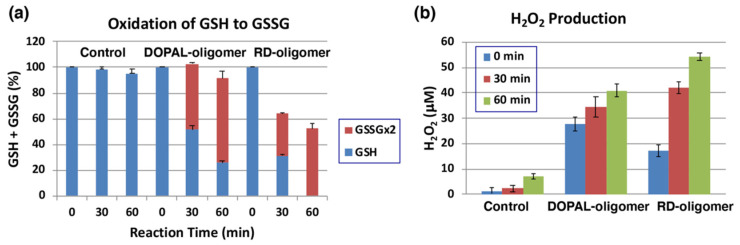
Pro-oxidant activity of DOPAL-oligomer. Oxidation of GSH and production of H_2_O_2_ by the DOPAL-oligomer and RD-oligomer (positive control). (**a**) The consumption of GSH and the production of GSSG from 1 mol eq. GSH. (**b**) the production of H_2_O_2_ before and 30 and 60 min after the oxidation of GSH. Mean ± SEM from three independent experiments (**a**,**b**).

The oxidation of 1 mM DOPAL by mushroom tyrosinase (100 U/mL) was carried out for 120 min at pH 7.4 and 37 °C to prepare DOPAL-oligomer. RD-oligomer was used as a positive control. Each of the oligomers was exposed to 1 mol equiv. of GSH for up to 60 min, and the remaining GSH and the oxidized glutathione (GSSG) were analyzed by specific HPLC methods [50]. As shown in Figure 7a, GSH levels decreased by ca. 50 to 70% during the first 30 min of incubation with the DOPAL-oligomer or RD-oligomer. This decrease can be ascribed to two independent but competing reactions: one is the binding of GSH to the quinonoid oxidation products, DOPAL-oligomer and RD-oligomer, and the other is the oxidation of GSH to GSSG. In the DOPAL-oligomer, the latter reaction appears to proceed to the major extent, while in the RD-oligomer, both reactions proceed almost at the same rate. It is interesting to note that the oxidation to GSSG proceeded in DOPAL-oligomer significantly faster than in RD-oligomer (51% vs. 33%, *p* < 0.01). Finally, we examined whether or not H_2_O_2_ is produced during the oxidation of GSH by the oligomers. As shown in Figure 7b, 41 µM and 55 µM of H_2_O_2_ were produced from the DOPLAL-oligomer and RD-oligomer during the 60 min reaction with GSH, respectively. Interestingly, the production of H_2_O_2_ at 0 min was significantly greater in DOPAL-oligomer (28 µM vs. 17 µM, *p* < 0.05). These results confirmed the pro-oxidant activity of the DOPLA-oligomer, the activity being as strong as that of the RD-oligomer.

## 3. Discussion

In the present study, we have explored possible mechanisms of DOPAL neurotoxicity, focusing on the chemical reactivity of two electron oxidation product(s) of DOPAL. At pH 7.4, the primary two electron oxidation product appears to be the corresponding quinone methide which exhibits two major reactions. The first reaction is an intramolecular cyclization that produces the benzofuran derivative, DHBF (Figure 1). The second reaction is the side chain hydration that aromatizes the quinone methide back to the catechol generating DOPEGAL. At pH 7.4, we could hardly detect the *o*-quinone isomer. Even if it is formed, it will undergo instantaneous isomerization to quinone methide at basic conditions as quinone to quinone methide tautomerization is a base catalyzed reaction [19,20,30,31,32]. By altering the reaction pH towards acidic conditions, we could detect the DOPAL-quinone transiently; but it also exhibits rapid intramolecular cyclization producing the same benzofuran derivative, DHBF, like the DOPAC-quinone which produces 2,5,6-trihydroxybenzofuran [27,33]. Thus, irrespective of whether *o*-quinone or quinone methide is formed, both reactions will lead to the same bicyclic product, DHBF. The production of DOPEGAL is possible only for quinone methide tautomer. Therefore, its formation confirms the transient production of DOPAL-quinone methide. A similar conversion of DOPAC to 3,4-dihydroxymandelic acid (DOMA) through DOPAC-quinone methide has been previously reported [27,33]. Thus, oxidation of DOPAL by mushroom tyrosinase or by Cu(II) ions produces DOPEGAL through the addition of water molecule to the quinone methide intermediate. DOPEGAL, when oxidized to the *o*-quinone, undergoes a loss of formate ion yielding DHBAld, through a reaction called aldehyde deformylation [37]. A similar type of reactions is rare in biological systems but can be found in the process of demethylation of the steroid hormone estrogen [37,51]. The loss of formyl group does not occur directly on DOPAL, nor even on DOPEGAL, as the reaction requires the further oxidation of DOPEGAL to its quinone. DOPEGAL-quinone can readily lose the formyl group, yielding a very transient and highly unstable quinone methide that would rapidly aromatize and generate DHBAld as the end product. A similar conversion of DOMA to DHBAld has also been reported in the literature [23,25,35]. The aldehyde deformylation is also a base-catalyzed reaction, as evidenced by the much faster decay of DOPEGAL (detected as DOPEG) at pH 7.4 than at pH 5.3 (Figure 2c,d).

The production of DHBF and THBF during the tyrosinase-catalyzed oxidation of DOPAL is not surprising as the related DOPAC also has been shown to produce the bicyclic benzofuran derivative—2,5,6-trihydroxybenzofuran [27,33]. In the case of DOPAC, the bicyclic product could be isolated and characterized. But it is highly likely that DHBF and THBF once formed are rapidly oxidized to oligomeric products in the absence of AA. In this connection, it should be stressed that DHBF is structurally considered as the oxygen analog of 5,6-dihydroxyindole, the major intermediate of eumelanin synthesis. 5,6-Dihydroxyindole is well known for its extreme susceptibility to oxidation [52,53]. It is very likely that DHBF (and THBF) oxidation products augment the pro-oxidant activity of DOPAL-oligomer, as seen in this study (Figure 7).

The structure of DHBF and THBF are proposed based on the following observations. (1) These two products could not be detected in the tyrosinase oxidation mixtures of DOPAL after the NaBH_4_ reduction (Figure 2c,d), suggesting their high reactivities under oxidative conditions. (2) Earlier studies with tyrosinase-catalyzed oxidation of DOPAC reported the production of a related benzofuran derivative, 2,5,6-trihydroxybenzofuran with an absorption maximum at 320 nm [27]. Additionally, the commercially available 6-hydroxydopamine exhibits an absorption spectrum (a maximum around 290 nm) which is rather different from those of DHBF and THBF (Figure S2). (3) THBF elutes out faster than DHBF on HPLC, suggesting that THBF is more hydrophilic as compared to DHBF. (4) Under less reactive conditions at pH 5.3 (Figure 3b), the tyrosinase-catalyzed oxidation of DOPAL in the presence of AA only afforded THBF in trace amounts (<1% at 30 min). We are conducting further studies to trap/characterize these unstable intermediates, which will be reported in the future.

The mechanism of formation of DHBF (and THBF) is postulated in Figure S4. The mechanism involves intramolecular nucleophilic addition of the hydroxyl group of the hydrated aldehyde to the quinone-methide tautomer of DOPAL. It is known that DOPAL and its oxidation product exist mostly in the hydrate form [22]. Certainly, the identification of DHBF (and THBF) needs to be confirmed by physicochemical methods on the isolated compound(s).

In the present study, DOPEGAL is rapidly produced in good yields when DOPAL is oxidized by mushroom tyrosinase or Cu^2+^ ions. Much less is known about the neurotoxicity of DOPEGAL compared to DOPAL. However, it has been shown that as low as 6 µM of DOPEGAL kills PC-12 cells [36] and a recent study showed that DOPEGAL is toxic to locus coeruleus neurons in mice [54]. It should be stressed that DOPEGAL appears to be more reactive and toxic than DOPAL [55,56]. Regarding the possible cytotoxicity of DHBAld, another product of DOPAL oxidation, a recent study showed that DHBAld was toxic to B16F10 melanoma cells at a concentration of 30 µM [57]. This weak cytotoxicity suggests that DHBAld itself would not contribute much to the neurotoxicity of DOPAL.

The presence of tyrosinase in neurons has been a subject of conjuncture [58,59]. However, this issue is not a subject of this study. Copper ions are present at modest (50 µmol/kg wet tissue or 1–2 µmol/g dry tissue) levels in various brain regions and play important roles in pathogenesis of neurodegenerative diseases [60,61,62,63]. Therefore, the finding in this study that Cu^2+^ ions at as low as 3 µM effectively oxidized DOPAL is physiologically relevant.

DOPAL exerts multiple potentially harmful protein modifications, including quinone addition, oligomerization, aggregation, and misfolding [12]. Both *o*-quinone and aldehyde groups appear necessary for DOPAL (and DOPEGAL) to exert toxicity to neurons [12,13,14,15]. The aldehyde group of DOPAL (and DOPEGAL) has been shown to react with the amino group in the lysine residues of proteins through Schiff-base formation [5,10,16]. This reaction leads to modifications and aggregation of proteins, involving the formation of stable isoindole cross-linkage [17,18]. DOPAL (and DOPEGAL) also reacts with Cys through the aldehyde group to form a thiazolidine via a Schiff-base [64]. However, it is apparent that *o*-quinone group is generally much more reactive than aldehyde group [19]. Therefore, as long as DOPAL is oxidized in neuronal cells, it is the *o*-quinone group that reacts with proteins through the thiol group in the Cys residues by 1,6-Michael type addition reaction [19]. The present study shows that DOPAL-quinone reacts effectively not only with a small thiol Cys to form 5-*S*-Cys-DOPAL and 2,5-*S*,*S*-di-Cys-DOPAL but also with protein thiol group in BSA. This is in line with the findings that *N*-acetylcysteine attenuates or prevents these modifications [12,65]. It has been shown that *N*-acetylcysteine dose-dependently suppressed α-synuclein modification (quinonization) by DOPAL induced by tyrosinase or Cu(II) [10,12]. This suggests that under oxidative conditions, the protein modifications result from the interaction with the *o*-quinone group but not with the aldehyde group in DOPAL. Jinsmaa et al. [10] showed that Cu(II)-catalyzed oxidation of DOPAL induces quinonization of α-synuclein more effectively than DA. This might contradict with our finding that the reactivity of DOPAL-quinone with BSA is at a similar level as DA-quinone. However, our results also showed that DOPAL is oxidized 5-times faster by Cu(II) than DA. Correctively, DOPAL should be more reactive (neurotoxic) than DA under oxidative conditions. The potent pro-oxidant activity of DOPAL-oligomer observed in this study adds to the toxic effects of DOPAL.

## 4. Materials and Methods

### 4.1. Materials

DOPAL was obtained from Cayman Chemical (Ann Arbor, MI, USA), and 6-hydroxydopamine, tyrosinase (from mushrooms, specific activity 1715 or 2687 U/mg), and Ampliflu^TM^ Red reagent (1-acetyl-3,7-dihydroxyphenoxazine) were purchased from Sigma-Aldrich (St. Louis, MO, USA). DOPEGAL was prepared by incubating 100 µM NE (200 µL) with 120 µg/mL human monoamine oxidase (recombinant, Sigma-Aldrich) in 50 mM sodium phosphate buffer (pH 7.4) at 37 °C. After 30 min of the reaction, 47% of NE was converted to DOPEGAL. The reaction was stopped by 0.8 M HClO_4_ (200 µL) and the mixture was used as a standard of DOPEGAL. As DHBAlc was not commercially available, a standard solution of DHBAlc was prepared by reducing 100 µM DHBAld (200 µL) in 50 mM sodium phosphate buffer (pH 7.4) with 10% NaBH_4_ (20 µL) followed by addition of 0.8 M HClO_4_ (200 µL). Standard solutions (100 µM) of Cys-DA, Cys-DOPET and Cys-DOPEG isomers were prepared by oxidizing 100 µM DA, DOPET or DOPEG by mushroom tyrosinase (50 U/mL) in the presence of 200 µM L-cysteine (Cys) in 50 mM sodium phosphate buffer (pH 6.8) at 25 °C. After 5 min, the oxidation was stopped by adding 10 µL of 6 M HCl to 1 mL reaction mixture. All other chemicals were of the highest purity commercially available. A stock solution of DOPAL was prepared at a 10 mM concentration in ethanol, which was diluted with buffer when necessary. The highest purity Milli-Q water (Milli-Q Advantage, Merck Millipore Co., Tokyo, Japan) was used throughout this study to avoid contamination of metal ions. The number of thiol group in BSA was determined to be 0.30 mol/mol protein [47].

### 4.2. Analytical Methods

HPLC analysis of the DOPAL reactions were carried out with a HPLC system consisted of a JASCO 880-PU pump (JASCO Co., Tokyo, Japan), an Osaka Soda C_18_ column (Capcell Pak MG; 4.6 × 250 mm; 5 µm particle size, Osaka Soda, Osaka, Japan) and a JASCO UV detector (JASCO Co., Tokyo, Japan) at 280 nm for catechols and 292 nm for Cys-catechols. The mobile phase consisted of 0.4 M formic acid: methanol, 90:10 (*v*/*v*) for the analysis of DOPAL oxidation products. HPLC analyses were performed at 35 °C for DOPET, DOPEG, and DHBAlc and at 50 °C for Cys-adducts of DOPET, at a flow rate of 0.7 mL/min. For analytical separation of HCl hydrolysis products of Cys-DOPET, a mobile phase of 0.4 M formic acid: methanol, 70:30 (*v*/*v*) was used at 50 °C with a flow rate of 0.7 mL/min. For preparative separation of the HCl hydrolysis products of Cys-DOPET, an Osaka Soda C_18_ preparative column (Capcell Pak MG; 20 × 250 mm; 5 µm particle size) was used at 50 °C and at a flow rate of 7.0 mL/min with a mobile phase of 0.4 M formic acid: methanol, 50:50 (*v*/*v*).

UV-visible spectra were analyzed with a JASCO V-630 UV-VIS spectrophotometer (JASCO Co., Tokyo, Japan). ^1^H NMR (400 MHz) spectra were obtained in 1 M DCl using a Bruker AVANCE 400 spectrometer (Billerica, MA, USA). High-resolution mass spectra were obtained using a 6220 TOF mass spectrometer (mode: electrospray ionization—time-of-flight, positive; ESI(+)-TOF) (Agilent Technologies, Santa Clara, CA, USA).

### 4.3. Oxidation of DOPAL by Tyrosinase or Metal Ions in the Absence or Presence of L-Ascorbic Acid (AA) or Cys

A solution (2 mL) of 100 μM DOPAL was oxidized by mushroom tyrosinase (50 U/mL) at 37 °C in 50 mM sodium phosphate buffer (pH 7.4 or 5.3). Changes in absorption spectra were periodically followed for 60 min using ultraviolet and visible spectrophotometer. The oxidation was also carried out in the presence of 1 mM ascorbic acid (AA) or 200 µM Cys. For the spectrophotometric analysis, the reference cell contained the same concentrations of buffer and tyrosinase. Aliquots of the reaction mixtures were also subjected to HPLC analysis. For reductive termination of the reaction, 200 μL aliquots were mixed with 20 μL 10% NaBH_4_, followed by 200 μL 0.8 M HClO_4_. For experiments in the presence of AA, the addition of NaBH_4_ was omitted. The above oxidation studies were also conducted with Cu^2+^, Fe^2+^, or Fe^3+^ ions replacing tyrosinase in the reaction mixture.

### 4.4. Oxidation of DOPAL, DOPET, or DA in the Presence of BSA and HPLC Estimates of Binding through the Cys Residue

The method developed for estimating the binding of *o*-quinones with proteins through the cysteinyl residues was used for the examining the reactivity of DOPAL also [34,43,44,66]. Typically, a solution (1 mL) of 100 μM DOPAL or other catechols and 300 µM BSA was oxidized by mushroom tyrosinase (50 U/mL) at 37 °C in 50 mM sodium phosphate buffer (pH 7.4) with gentle shaking. After 5 min of reaction, 100 μL of each reactant was added to a 10-mL screw-capped conical glass test tube containing 10 µL of 10% NaBH_4_, which was followed by the addition of 10 µL of 6 M HCl and 30 µL of water. After vortex mixing, 500 µL of 6 M HCl containing 5% thioglycolic acid and 1% phenol was added, and the mixture was hydrolyzed for 20 h at 110 °C under an argon atmosphere. Thioglycolic acid and phenol are essential to protect catecholic products from oxidation. After cooling, 130 μL of each reaction mixture was transferred to 1.5-mL micro-tubes containing 50 mg alumina and 200 μL 1% Na_2_S_2_O_5_-1% disodium EDTA. To this, 700 μL 2.7 M Tris-HCl (pH 9.0)-2% disodium EDTA was added and shaken vigorously for 5 min, followed by centrifugation for 15 s at 10,000× *g*. The upper layer was removed using an aspirator and the alumina was washed with 1 mL of Milli-Q water followed by aspiration of the upper layer after centrifugation for 15 s at 10,000× *g*. This washing procedure was repeated three times. Finally, Cys-catechol isomers bound on alumina were extracted by shaking the alumina vigorously with 200 μL of 0.4 M HClO_4_ for 2 min followed by centrifugation for 15 s at 10,000× *g*. The Cys-catechol isomers thus isolated were analyzed by HPLC. For the standard Cys-catechol isomers, 100 μL of 100 µM standard solution containing Cys-catechol isomers (for Cys-DA isomers, 5-*S*-Cys-DA, 2-*S*-Cys-DA, and 6-*S*-Cys-DA) were added to a 10-mL screw-capped conical glass test tube containing 10 μL of 10% NaBH_4_ followed by the addition of 10 µL of 6 M HCl and 30 µL (30 nmol) of BSA. After vortex mixing, 500 µL of 6 M HCl containing 5% thioglycolic acid and 1% phenol were added, and the mixture was hydrolyzed for 20 h at 110 °C under an argon atmosphere. After cooling, Cys-catechol isomers were isolated and analyzed in the same way as described above. The yields of Cys-catechol isomers were obtained by comparing peak areas to the standard. Retention times of three major products were 6.3 (the original Cys-DOPET), 11.2, and 17.7 min, in a typical HPLC chromatogram of total Cys-DOPET-derived products from hydrolysates of DOPET-BSA conjugate.

### 4.5. Synthesis of Cys-DOPET and Cys-DOPET Derivatives

A solution of 32.9 mg (0.214 mmol) of DOPET and 51.8 mg of Cys (0.428 mmol) in 21 mL of 50 mM sodium phosphate buffer (pH 6.8) was oxidized by mushroom tyrosinase (7.9 mg, 2687 U/mg) at 25 °C. After 60 min of gentle stirring, the oxidation was stopped by the addition of 0.2 mL of 6 M HCl. HPLC analysis of the reaction mixture indicated the complete disappearance of DOPET with its conversion to Cys-DOPET isomers. The Cys-DOPET isomers were isolated by ion exchange chromatography on Dowex-50W-X2 (1.6 cm × 6 cm in water). The column was washed with 50 mL of 0.5 M HCl, eluted with 2 M HCl and fractions of 10 mL were collected. Fractions containing the desired compound were evaporated to give 67.6 mg (91% yield) of Cys-DOPET·HCl·2H_2_O salt. HPLC analysis showed that the mixture contained the major isomer 5-*S*-Cys-DOPET (94%) with the minor isomer of 2-*S*-Cys-DOPET (6%). This mixture was subjected to the following reaction without further purification.

A solution of 32.9 mg (0.095 mmol) of the above mixture of 5-*S*-Cys-DOPET and 2-*S*-Cys-DOPET in 5 mL of 6 M HCl containing 5% thioglycolic acid and 1% phenol was heated for 20 h at 110 °C. HPLC analysis showed a conversion of Cys-DOPET isomers to two more lipophilic products. The mixture was evaporated to remove HCl and then subjected to Dowex 50W-X2 chromatography (1.6 × 4 cm in water). The column was washed with 20 mL of 0.1 M HCl, eluted with 3 M HCl and fractions of 10 mL were corrected. Fractions containing Cys-DOPET derivatives were evaporated to give 21.0 mg of solids. Preparative HPLC of the Cys-DOEPT derivatives afforded three products, unchanged Cys-DOPET and two new derivatives, Cys-DOPET-SCH_2_COOH and Cys-DOPET-Cl, in the sequence of retention time. Cys-DOPET-SCH_2_COOH and Cys-DOPET-Cl are those derived from substitution of the alcoholic hydroxyl group by carboxymethylthio (-SH_2_COOH) group and chloride atom, respectively. Their amounts and yields (and HPLC purity) were 5.1 mg in 20% (purity 98%), 4.8 mg in 15% (purity 93%), and 6.5 mg in 23% (purity 98%) for the original Cys-DOPET, the carboxymethylthio derivative, and the chloride derivative, respectively. Their ^1^H-NMR and mass spectra are summarized in Table S1.

### 4.6. Pro-Oxidant Activity of the DOPAL Oxidation Product, DOPAL-Oligomer

DOPAL-oligomer and RD-oligomer were prepared in 50 mM sodium phosphate buffer (pH 7.4) from the precursors DOPAL (1 mM) and RD (1 mM) as previously described [34,46,47,48]. Tyrosinase (200 U) was added to 2 mL of each precursor solution and the mixture was incubated at 37 °C for 120 min with vigorous mixing. Tyrosinase alone (100 U/mL) was used as a control. The oligomer solutions (2 mL) were mixed with 10 mM GSH (200 µL, 1 mol eq.) and were incubated at 37 °C. At 0-, 30- and 60-min reaction times, 100 µL aliquots were withdrawn and mixed with 0.4 M HClO_4_ (800 µL) to terminate the oxidation. GSH and GSSG in the oxidation mixtures were analyzed using our HPLC method [34,46,47,48,50]. H_2_O_2_ in the oxidation mixture (20 µL) was analyzed spectrophotometrically after dilution with the pH 7.4 buffer (180 µL). The diluted mixture was reacted with the chromogen Ampliflu^TM^ Red reagent (200 µL) to form a red pigment having an absorption maximum at 568 nm [67] closely following the manual (Invitrogen, Tokyo, Japan).

### 4.7. Statistical Analysis

Students’ *t*-test employed with JMP 10 software (SAS Institute Inc., Cary, NC, USA). *p* values <0.05 were considered significant with one-tailed analysis.

## 5. Conclusions

Tyrosinase-catalyzed oxidation of DOPAL produced both DOPAL-quinone and tautomeric quinone-methide, which gave rise to DOPEGAL and DHBAld. Two additional cyclized products—DHBF and THBF could be detected in the reaction mixture under reducing conditions. Physiological concentrations of Cu(II) ions could also generate quinonoid products in the reaction mixture, which caused protein binding, pro-oxidant activity and reactive oxygen species production. These results indicate that the oxidation products of DOPAL exhibit several toxic effects augmenting the neurotoxicity of DOPAL.

## Supplementary Materials

The following are available online at https://www.mdpi.com/article/10.3390/ijms222111751/s1.

## Abbreviations

AA	ascorbic acid
Cys	L-cysteine
DA	dopamine
DHBAlc	3,4-dihydroxybenzylalcohol
DHBAld	3,4-dihydroxybenzaldehyde
DHBF	5,6-dihydroxybenzofuran
DOMA	3,4-dihydroxymandelic acid
DOPA	L-3,4-dihydroxyphenylalanine
DOPAC	3,4-dihydroxyphenylacetic acid
DOPAL	3,4-dihydroxyphenylacetaldehyde
DOPEGAL	3,4-dihydroxyphenylglycolaldehyde
DOPET	2-(3,4-dihydroxyphenyl)ethanol
DOPEG	3,4-dihydroxyphenylethylene glycol
MAO	monoamine oxidase
NE	norepinephrine
RD	rhododendrol
THBF	3,5,6-trihydroxybenzofuran
