# Supplementary material for: Oxidative Transformations of 3,4-Dihydroxyphenylacetaldehyde Generate Potential Reactive Intermediates as Causative Agents for Its Neurotoxicity
Source: Int J Mol Sci. 2021 Oct 29;22(21):11751. doi: 10.3390/ijms222111751 (PMC8583873; doi:10.3390/ijms222111751)
Supplement: Supplementary file 1 [file ijms-22-11751-s001.zip › ijms-1423422-Supplemental Materials.pdf]

Supplemental Materials

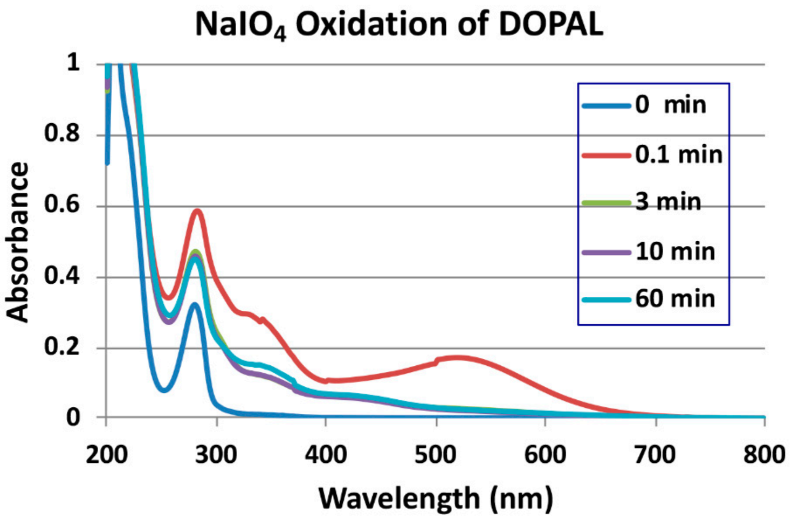

Figure S1. Time course of the oxidation of DOPAL (100 μM) by sodium periodate (100 μM) at pH 7.4 and 25°C. The absorption maximum of 0.173 (78% of 0.223) at 520 nm after 0.1-min oxidation was lower than that in the tyrosinase-catalyzed oxidation (0.223 at 518 nm). This can be explained by incomplete oxidation of DOPAL (23% remaining by HPLC) in the sodium periodate oxidation. Data were obtained from a single experiment, but reproducibility was confirmed.

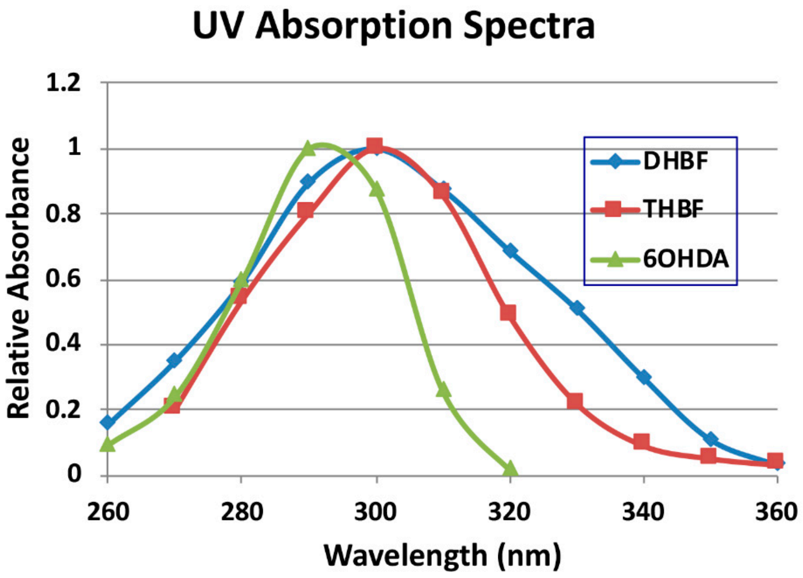

Figure S2. UV spectra of DHBF, THBF, and 6-hydroxydopamine (6OHDA) determined on HPLC peak heights. Peak heights were normalized for those at the maxima at 300 nm (for DHBD and THBF) or at 290 nm (for 6OHDA). Averages for two independent determinations.

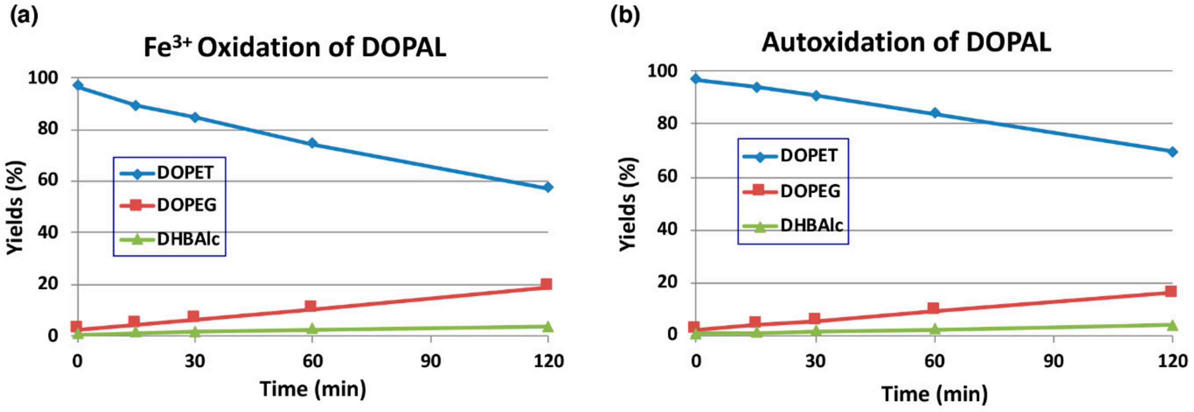

Figure S3. Time course of HPLC following of Fe^{3+}-catalyzed or autoxidation of DOPAL (100 μM). (a) Fe^{3+} (100 μM) at pH 7.4 and 37°C. (b) air at pH 7.4 and 37°C. Reaction was stopped by the addition of $NaBH_4$ followed by 0.8 M $HClO_4$. Data were obtained from averages of two independent experiments.

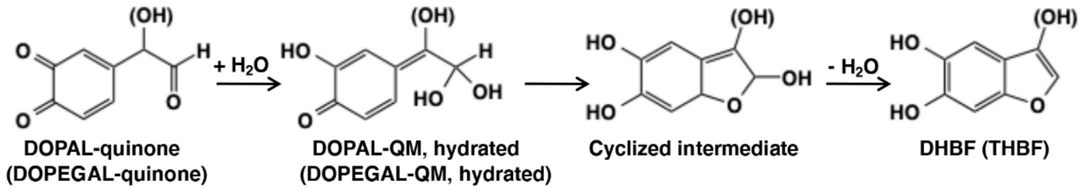

Figure S4. Postulated mechanisms for the production of DHBF and THBF from DOPAL-quinone and DOPEGAL-quinone.

Table S1. ^{1}H-NMR and mass spectra of Cys-DOPET, carboxymethylthio (CMT)-Cys-DOPET, and chloro-Cys-DOPET[a]

Position	Cys-DOPET	CMT-Cys-DOPET	Cl-Cys-DOPET
H-4	6.95 (1H, d, 1.6 Hz)	6.96 (1H, d, 1.6 Hz)	6.98 (1H, d, 2.0 Hz)
H-6	6.84 (1H, d, 1.6 Hz)	6.84 (1H, d, 1.6 Hz)	6.87 (1H, d, 2.0 Hz)
H-1'	2.70 (2H, t, 6.4 Hz)	2.78 (2H, m)	2.93 (2H, t, 6.8 Hz)
H-2'	3.76 (2H, t, 6.4 Hz)	2.86 (2H, m)	3.77 (2H, t, 6.8 Hz)
$SCH_2C\underline{H}$	4.22 (1H, m)	4.22 (1H, m)	4.21 (1H, t, 5.6 Hz)
$SC\underline{H}_2CH$	3.45 (2H, m)	3.45 (2H, m)	3.46 (2H, d, 5.6 Hz)
$SC\underline{H}_2COOH$		3.33 (2H, s)	

[a] The spectra were taken in 1 M DCl. Chemical shifts are shown in ppm relative to the solvent sygnal at 5.191 ppm (HDO).

High resolution mass spectral analyses: Cys-DOPET [3-*S*-cysteinyl-5-(2-hydroxyethyl)-catechol], 274.0747 $(M+H)^+$, Calc'd for $C_{11}H_{16}O_5NS$ 274.0750; Cys-DOPET-SCH_2COOH [3-*S*-cysteinyl-5-(2-carboxymethylthio-ethyl)-catechol], 348.0584 $(M+H)^+$, Calc'd for $C_{13}H_{18}O_6NS_2$ 348.0576; Cys-DOPET-Cl [3-*S*-cysteinyl-5-(2-chloroethyl)-catechol], 292.0419 $(M+H)^+$, Calc'd for $C_{13}H_{18}O_6NSCl$ 292.0412. ^{35}Cl to ^{37}Cl ratio 72.8% to 27.2%. Natural abundance 75.8% to 24.2%.

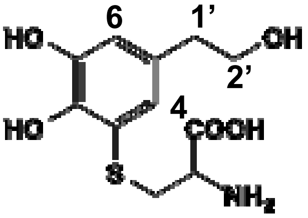

Cys-DOPET

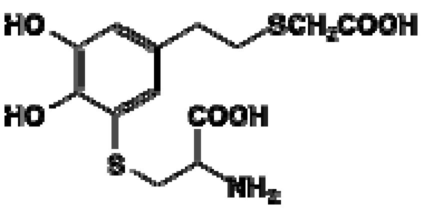

CMT-Cys-DOPET

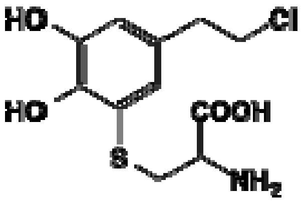

Cl-Cys-DOPET
